# Defining Precision Medicine Approaches to Autism Spectrum Disorders: Concepts and Challenges

**DOI:** 10.3389/fpsyt.2016.00188

**Published:** 2016-11-29

**Authors:** Eva Loth, Declan G. Murphy, Will Spooren

**Affiliations:** ^1^Department of Forensic and Neurodevelopmental Sciences, Institute of Psychiatry, Psychology and Neuroscience, King’s College London, London, UK; ^2^Sackler Institute for Translational Neurodevelopment, Institute of Psychiatry, Psychology and Neuroscience, King’s College London, London, UK; ^3^Roche Pharmaceutical Research and Early Development, NORD Discovery and Translational Area, Roche Innovation Center, Basel, Switzerland

**Keywords:** autism spectrum disorder, biomarkers, precision medicine

## Abstract

The tremendous clinical and etiological variability between individuals with autism spectrum disorder (ASD) has made precision medicine the most promising treatment approach. It aims to combine new pathophysiologically based treatments with objective tests (stratification biomarkers) to predict which treatment may be beneficial for a particular person. Here we discuss significant advances and current challenges for this approach: rare monogenic forms of ASD have provided a major breakthrough for the identification of treatment targets by providing a means to trace causal links from a gene to specific molecular alterations and biological pathways. To estimate whether treatment targets thus identified may be useful for larger patient groups we need a better understanding of whether different etiologies (i.e., genetic and environmental risk factors acting at different critical time points) lead to convergent or divergent molecular mechanisms, and how they map onto differences in circuit-level brain and cognitive development, and behavioral symptom profiles. Several recently failed clinical trials with syndromic forms of ASD provide valuable insights into conceptual and methodological issues linked to limitations in the translatability from animal models to humans, placebo effects, and a need for mechanistically plausible, objective outcome measures. To identify stratification biomarkers that enrich participant selection in clinical trials, large-scale multi-modal longitudinal observational studies are underway. Addressing these different factors in the next generation of research studies requires a translatable developmental perspective and multidisciplinary, collaborative efforts, with a commitment to sharing protocols and data, to increase transparency and reproducibility.

## Introduction

When parents first receive a diagnosis of autism spectrum disorder (ASD) of their child, some of their most pressing questions are: what is the prognosis of my child? What has caused his/her autism? And what are the treatment options?

Autism spectrum disorder is a clinically and etiologically heterogeneous condition, currently estimated to affect between 1 and 1.5% of children and adults worldwide (1, 2). Diagnostic ascertainment is based on the behavioral symptom profile alone; the co-occurrence of social-communicative deficits, repetitive and restricted behaviors and interests, and sensory processing anomalies [DSM-5 (3)]. In addition, up to 70% of individuals have one or more psychiatric and/or medical comorbidities, such as intellectual disability, ADHD, irritability, aggression, anxiety, depression, epilepsy, and sleep anomalies (4).

The prognosis is very variable. A recent large-scale longitudinal study showed distinct developmental trajectories in children between the ages of 2 and 14 years (5). Children whose symptoms were least severe at first diagnosis showed the most symptom improvement. However, a subgroup of around 10% of children who presented with the most severe social deficits at age 3 years made significant gains in their social trajectory across childhood. Nevertheless, for the majority of people with ASD, outcome in adulthood has been estimated to be “poor” (46%) or even “very poor” (12%) (6). IQ and language level are widely considered the best predictors of outcome. Beyond this, it is currently largely unknown whether different developmental trajectories may reflect different biological subgroups, and why some individuals develop comorbidities but others do not.

To date, no effective medical treatments are available that significantly improve the core symptoms of ASD. Only two medications (the second-generation antipsychotics risperidone and aripiprazole) have been approved in ASD by the US Food and Drugs Administration (FDA) and one (risperidone) by the European Medicines Agency (EMA). Both medications are not specific for ASD and target associated symptoms, such as aggression or irritability. Instead, the management of ASD relies heavily on behavioral and educational interventions (7). Although several of these programs report significant improvements, difficulties in generalizing skills to “real-world” settings, and access to these treatments and their expense, remain common limitations (8).

## The Call for a Precision Medicine Approach

Recognition of the phenotypic and etiological variability between individuals on the autism spectrum and the lack of effective treatments has called for a precision medicine approach. This approach aims to identify targeted treatments based on the understanding of the underlying pathophysiology and to then combine the drug (or intervention) with a companion diagnostic (stratification biomarker) to select or exclude patients for a particular treatment. Below we review current progress and discuss some of the challenges and requirements that still lie ahead.

Perhaps the biggest breakthrough for drug discovery in ASD came from the identification of syndromic and monogenic forms of ASD (where the disorder is thought to be caused by a highly penetrant single gene). Hundreds of ASD risk genes have been identified (9, 10), and more are expected to be found over the next years through whole-genome sequencing and studies with larger sample sizes. The significance of these discoveries lies in their potential ability to identify a causal link from a gene to cellular and molecular mechanisms underlying ASD symptoms (11). Moreover, although each of these monogenic forms is rare (i.e., found in less than 1% of individuals with ASD) different genes have been shown to converge on affecting a much smaller number of common pathways (12). This finding is crucial as it means that a particular biological pathway could be a treatment target rather than individual gene products. Treatments thus identified may therefore, potentially, be applicable for broader patient groups. Many of these risk genes modulate pathways involved in synapse formation and function, as well as other cellular functions, such as chromatin remodeling and transcription, protein synthesis and degradation, and receptor signaling (10). Any of these mutations may therefore alter essential developmental processes *in utero* or shortly after birth (13). For example, abnormalities in synapse development, function, and plasticity may broadly impact the balance between excitation (mainly modulated by glutamate) and inhibition (mainly modulated by gamma-aminobutyric acid, GABA) In particular, it has been suggested that (some forms of) ASD may be linked to disproportionally high levels of excitation and cortical network function (14).

Animal models of syndromic and monogenic forms showed significant pre-clinical promise such that several molecular aberrations and behavioral phenotypes could be reversed through pharamacological treatment or genetic rescue – sometimes even in adulthood (15, 16). Subsequent Phase I clinical trials using mGluR antagonists, or a GABA_B_ agonist, also reported promising results.

However, well-powered (Phase IIb or Phase III) double-blind placebo-controlled clinical trials with individuals with Fragile X syndrome so far produced disappointing results (17). For example, two clinical trials, led by Roche and Novartis, respectively, reported a lack of efficacy of their mGluR-inhibiting drugs (RG7090 and mavoglurant) in Fragile X syndrome. Similarly, a trial with arbaclofen, led by Seaside Therapeutics, failed to find significant improvements in individuals with Fragile X and idiopathic autism relative to placebo. This highlights several factors.

First, even monogenic or syndromic forms also involve considerably heterogeneous symptom expression. For example, although a SHANK3 haploinsufficiency (thought to cause Phelan McDermid Syndrome) is one of most penetrant genetic risk factors for ASD [approximately 70% (18)], it can also lead to ADHD, schizophrenia, or bipolar disorder in a smaller number of individuals (19). About 30% of people with Fragile X syndrome have ASD (20). Others present with ADHD, anxiety and avoidance behavior, mood instability, or aggressive behavior (21). This variable expressivity appears to be not solely explained by the size or location of the deletion alone. What are the factors that may influence shared vs. distinct developmental outcomes; and at what level(s) can divergence or variability be observed?

## Factors That Impact Developmental Outcome

**Figure 1:**
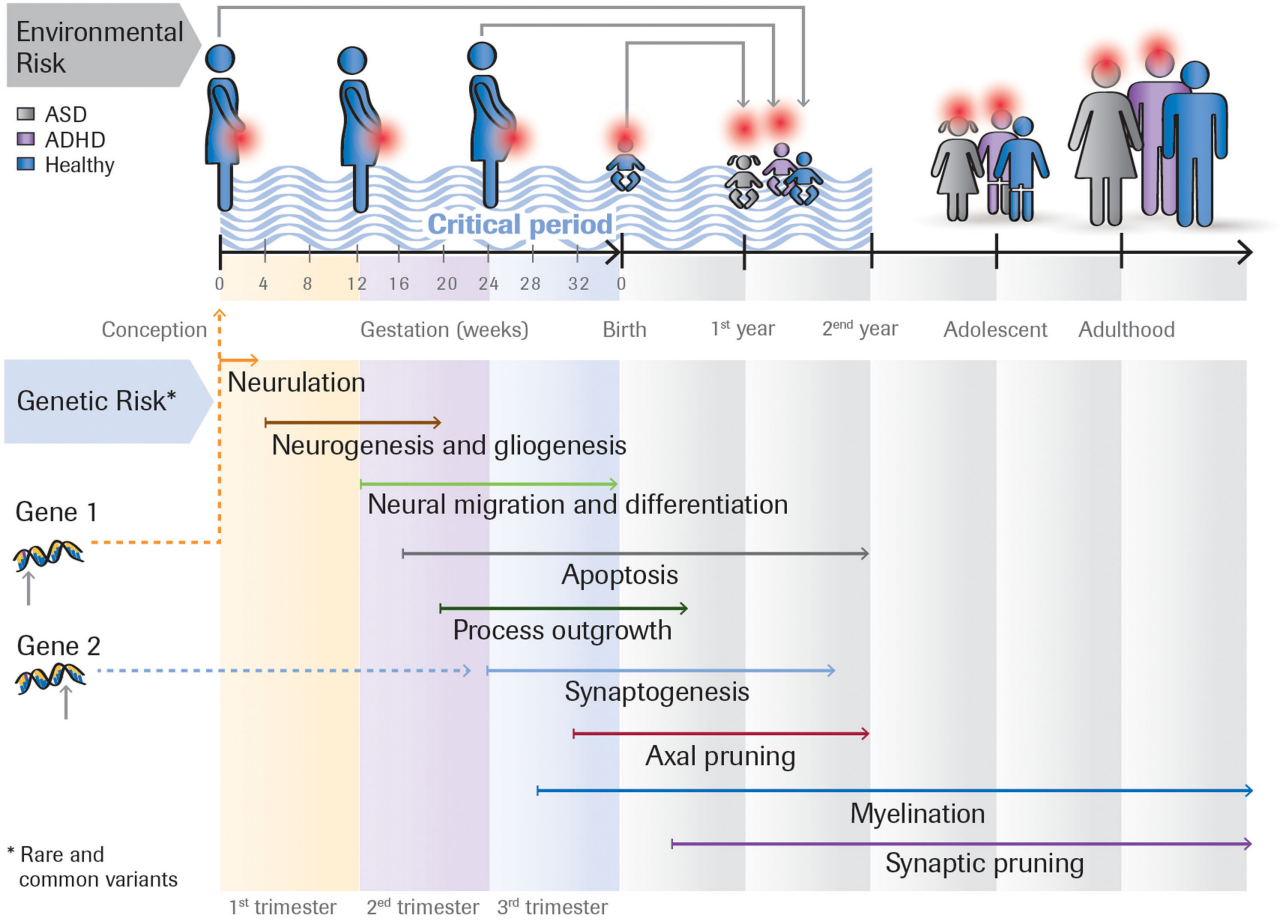
**Rare and common genetic variants, as well as environmental risk factors (e.g., toxins, maternal infection) acting at different time points impact on the developmental outcome**. For example, the same rare genetic mutation can either lead to ASD, ASD + ADHD, or ADHD, depending on the person’s genomic background (the sum of common variants). Or the effect of a genetic variant could be modulated by environmental insults that occur during the first, second or third trimester of the pregnancy. Many ASD risk genes are involved in essential developmental processes including proliferation, neuronal growth and differentiation, and synapse formation. These genes are expressed at different times in brain development.

Figure 1 outlines the interplay between four factors that may impact on developmental outcome.

### Common Variants and Genomic Background

The impact of rare mutations on phenotypic outcome may depend on genomic background (the sum of common variants). It may act as a “buffer” that diminishes or increases the deleterious effect of rare variants (CNVs) – for example, by modulating synaptic homeostasis (10). For an individual with a genetic background that contains a high load of common ASD risk variants, a small burden of rare risk genes may suffice to cause ASD. By contrast, an individual whose genetic background only includes a small number of common risk variants may require a higher burden of deleterious mutations to cause ASD. The rare genetic mutation may still have some penetrance, but the effect may be “milder,” including sub-threshold clinical symptoms (10).

### Environmental Risk Factors

In addition to genetic factors, it is likely that environmental influences – notably those acting during the embryonic stage – modulate risk for ASD. This includes maternal infections and prenatal exposure to teratogenic agents such as valproate acid, exposure to toxins, and dysfunction of the immune system (22). For instance, human studies comparing monozygotic and dizygotic twins have consistently revealed significant environmental influences that account for approximately 5–6% (23) of the observed variance in ASD. Moreover, higher concordance rates of ASD and other neurodevelopmental disorders in dizygotic twins than (singleton) siblings suggest a specific role of the fetal environment because both the twins and singletons share 50% of their genes (24).

### Critical Periods

The time when different factors impact on neuronal development likely plays a crucial factor in determining developmental outcome. Brain development is initially determined by distinct temporal and spatial stages of gene expression (25) and intrinsic neuronal activity (26) but then becomes actively refined by interactions with the environment. Timing could be influenced either by genetic factors as different genes are expressed at different times in brain development [e.g., in humans genes involved in cell proliferation are expressed earlier than those involved in synaptogenesis or myelination (25)] or the time when an environmental insult occurs.

By altering the developmental window during which genetic/environmental insults are applied, animal studies can trace these effects across cellular, molecular, brain systems, and behavioral levels. However, the vast majority of animal studies have only tested adult animals. Therefore, we only know the end state – and not how abnormalities developed or changed across development. To understand this is vital because it is possible that some treatment effects may be different in developing vs. adult brains. A treatment that is likely only effective in early development would raise important ethical implications for clinical trial designs that usually first test safety, efficacy and side-effects in adults.

## Translatability

One potential factor in failed clinical trials could be limited translatability from animal models to humans. Patient-derived induced pluripotent stem cells (iPSCs) are an interesting new approach that overcomes inter-species differences, although the technology is still in need of further development. Recently, protocols have been developed to derive iPSCs from peripheral blood mononuclear cells (27) or hair roots (28). This makes sample collection significantly easier and more viable for vulnerable and larger patient groups than earlier protocols based on fibroplasts (derived from skin biopsies). In addition, the ability to freeze keratinocytes themselves now offers great flexibility in generating lines from patients with particular genetics and phenotypic characteristics. For example, by comparing lines from patients with a particular monogenic defect (e.g., with SHANK3), but different clinical symptoms we may be able to account for differences in genomic background (which is difficult in animal models) and identify which cellular alterations may be linked to the gene vs. particular phenotypic differences. Comparison of phenotypes between cells derived from patients with monogenic vs. “idiopathic” forms of ASD provides valuable information on how generalizable cellular or molecular alterations identified from specific genetic/environmental processes may be for wider patient groups. Currently, the iPSC methodology is very costly. However, by studying cell lines of patients that are also comprehensively characterized in terms of their systems level (MRI, EEG, and PET) and neurocognitive profile we will be able to advance our understanding of the relationship between cellular, morphological and molecular, and higher level phenotypes in the same person. New systems level features of brain anatomy, function, and connectivity are now developed that offer higher resolution and greater translatability to brain phenotypes studied in animal models (29).

## We Need to Bridge Levels of Analyses – Cognition as a Black Box

One of the current challenges for neurobiological hypotheses, such as the E/I imbalance hypothesis, lies in its broadness: both glutamate and GABA are ubiquitous in the brain. Is the E/I imbalance in ASD cell and/or circuit-specific? Or might phenotypic differences linked to E/I imbalances across disorders (e.g., ASD vs. schizophrenia, ID) depend on a critical period during which they occur? And how do E/I imbalances give rise to characteristic cognitive profiles of ASD that involves both weaknesses and strengths?

The immature brain undergoes progressive alterations in molecular composition and in synchronized currents that underpin the development of functional neuronal circuits. Synchronized patterns of neuronal activity engage many neurons of developing networks, possibly because of efficient feed-forward GABA-ergic inhibition. These immature signals stop at critical time points to enable behaviorally relevant brain activity to emerge, which requires sparse fired, time-locked oscillations. Whereas early perturbations during basic circuit refinement may lead to widespread abnormalities, later occurring ones may produce more specific and localized disruptions. Brain networks may also differ in their resilience to gene dosage such that the functional effects of abnormal gene dosage could be localized even if the genetic abnormalities are widespread (30). Brain networks involved in evolutionarily older biological processes are thought to have developed more compensatory mechanisms than those supporting more recent cognitive functions. For example, the timing of insults in synapse development differentially affects different cortical regions as the timeline for synaptogenesis is different across the cortex (31). Changes in synaptic function and timing might then particularly disrupt the connectivity of higher order association areas, including frontal–parietal, frontal–temporal, and frontal–striatal circuits (32). This coincides with brain systems supporting higher level social-cognitive function or language development, which spike in synaptogenesis and plasticity between 1 and 3 years of age (31); roughly the time when social and language-related symptoms often become first apparent in ASD. Transcriptomics studies of co-expression patterns showed enrichment of ASD genes in cortical projection neurons (33), including glutamatergic projection neurons in superficial cortical layers (34).

Recently, some efforts were also made in linking glutamate or GABA neurotransmission to sensory processing abnormalities. For example, deficits in binocular rivalry (35) as well as paradoxical motion perception (36) in ASD are taken as indirect proxies for reduced GABA-ergic signaling. However, this approach makes the strong assumption that anomalies in circuits underpinning sensory anomalies are “primary deficits” in autism (37, 38) with down-stream effects on networks supporting higher level (and later developing) cognitive and social-cognitive functions – a premise that remains to be further tested.

Finally, some brain circuits may be more affected than others because glutamate and GABA modulate and are modulated by other neurotransmitter systems in particular brain regions and that are crucial for cognitive functions. For example, at birth, GABA-ergic signaling shifts from excitation to inhibition due to a reduction in intracellular chloride concentration, which in turn is mediated by endogenous oxytocin release. This shift in GABA-ergic polarity is abolished in mouse models of fragile X syndrome and rodents treated with valproate *in utero*; and some evidence indicates that “immature” excitatory GABA-ergic activity may persist in people with ASD (39). This provides a potential link of GABA to a cascade effect of social-motivational abnormalities that are thought to be modulated by oxytocin.

### Well-Validated Translated Cognitive Tests for Large-scale Investigations

Cognitive measures that map onto specific circuits are needed to bridge our understanding between systems level and behavioral anomalies. For many commonly used tests, psychometric properties (e.g., test–retest reliability), age norms, are not available. We need to invest in well-validated cognitive batteries that are equally suitable and meaningful for both children and adults, or for which different comparable versions exist. As most syndromic and monogenic forms of ASD involve varying degrees of intellectual disability, these tests should also be sensitive across the ability ranges, including profound intellectual disability levels to understand whether mechanisms are shared or different between these forms of ASD.

## What are Mechanistically Plausible Clinical Endpoints?

International regulators, such as the FDA, require clinical trials to select only one assessment as the primary endpoint against which the success of a study is measured. Currently, the lack of a truly mechanistic understanding of the link between molecular, neuroanatomical/functional, and cognitive processes impedes informed selection of “primary endpoints.” For example, in a pilot trial of insulin-like growth factor-1 (IGF-1) in Phelan McDermid Syndrome the Aberrant Behavior Checklist (ABC) social withdrawal subscale was chosen as the primary outcome measure because it is well validated in ASD and ID and accepted within pediatric psychopharmacology research (40). In the STX209 arbaclofen trial with volunteers with “idiopathic” ASD, the ABC-irritability subscale was used as the primary outcome measure; again because the measure is known to be sensitive to change in pharmacologic trials. However, in both examples there was no mechanistic reason why the growth-stimulating hormone should primarily affect social symptoms or a specific GABA_B_ receptor agonist should “specifically” affect irritability (rather than core social deficits of ASD). Also, in some instances, the clinical outcome measure (irritability) did not directly translate to the most consistent behavioral changes identified in the mouse model (learning) (17).

## Placebo Effects

Placebo effects are a major difficulty for testing treatment efficacy in double-blind randomized control trials, which result from participants’ expectation that they are receiving a treatment and may lead to conscious or unconscious behavioral changes. Even the support, attention, and interest from the research team compared to the person or family’s everyday experience may have a “therapeutic” effect. The effect size of the placebo response in medication trials for ASD is estimated to be “moderate” (41). Recently, trial designs have been proposed that aim to address the interaction between those behavioral changes and treatment (42). In addition, demonstration of target engagement using objective measures may be one way forward to evaluate the efficacy of the treatment in clinical trials, even when – by virtue of the placebo effects – changes in overall outcome may not be significantly different between the treatment and control groups.

## Stratification Biomarkers

The next important step is to select particular patients groups for clinical trials that are more likely to respond to the treatment under consideration. Despite an explosion of biomarker research over the past years, we currently do not have a single validated or clinically useful biomarker for ASD. Biomarkers have been defined as “a characteristic that is objectively measured and evaluated as an indication of normal biological processes, pathogenic processes, or pharmacologic responses to a therapeutic intervention” (43). In the past, using case–control designs, the majority of research studies focused on the identification of *diagnostic biomarkers* to provide a discrete and objective indication of diagnostic status (i.e., whether or not someone has an ASD). Although many studies reported significant *mean group* differences in, for example, performances on a range of cognitive tests, or in brain structure, function, or connectivity (using MRI methods), inconsistencies in findings and failure to replicate are common problems. Moreover, it is important to note that a mean group difference alone (especially with moderate effect sizes) by no means indicates that that measure has diagnostic biomarker utility. This further requires an almost complete non-overlap of the distributions of individuals in the case and control groups (and, according to traditional categorical classifications, individuals with other neurodevelopmental or neuropsychiatric conditions).

*Stratification biomarkers* divide a group of patients into subgroups with shared biological characteristics. These subgroups may differ in terms of their clinical symptom profile and/or etiology. Stratification markers may be primarily clinically relevant if they have either *prognostic* value, i.e., they assess the (untreated) progression and outcome of the disorder, or *predictive* value, i.e., they estimate the probability of response to a given treatment. Stratification biomarker research in ASD is still in its infancy. Difficulties for stratification research have been primarily studies with small sample sizes, such that the majority of cognitive or neuroimaging studies include 15–30 participants per group – which are associated with limited power, especially if a group were divided into two or more subgroups.

Large-scale multi-center multidisciplinary observational studies, such as the EU-AIMS Longitudinal European Autism Project (LEAP) are currently underway that have sufficient power to identify stratification markers (44). Multi-modal assessments of each individual allow us to identify genetic, molecular, circuit-based, and behavioral markers.

One approach to stratification is to split the sample based on *a priori* participant criteria (e.g., sex, developmental level). For example, there is some evidence that females with ASD differ from males with ASD in terms of their cognitive profile, neuroanatomy, or function (45) and that females may require a higher burden of genetic risk factors to develop ASD [i.e., being more protected from developing ASD (46)]. There may also be differences between individuals with ASD with/without distinct co-occurring conditions, such as ADHD or anxiety. In addition, unsupervised, data-driven methods may be particularly useful to identify subtypes based on differences in, for example, brain anatomy, function, and/or cognitive profile. To do this, hierarchical clustering methods (47) or normative modeling approaches to neuroimaging data (48), have recently been used. Larger-scale neuroimaging studies of ASD [e.g., EU-AIMS LEAP (44); Province of Ontario Neurodevelopmental Disorders Network, POND (49)] or efforts to aggregate neuroimaging data from different laboratories [Autism Brain Image Data Exchange, ABIDE (50)] now begin to provide cohorts of sufficient sizes and to enable replication. In contrast to the relative high costs of MRI scans, electrophysiological methods are relatively less expensive, and easy to use in young children and even infants, and individuals with intellectual disabilities (51). Valid EEG stratification markers may therefore be in principle more feasible to implement in clinical practice. Many circuits underpinning fundamental bio-behavioral dimensions affected in ASD (e.g., social cognition, reward processing) cut across different neurodevelopmental and neuropsychiatric disorders. Therefore, a biomarker that estimates deficits in, for example, (neural activation underlying) emotional reactivity or reward sensitivity does not necessarily have to be specific to ASD but instead may predict dimensional symptom severity (52).

Multi-modal biomarkers (e.g., combining resting-state EEG and fMRI) likely have improved prognostic as well predictive value relative to markers based on one modality. For example, abnormalities in gamma band oscillations could indicate either increased excitatory (e.g., glutamatergic) or reduced inhibitory (e.g., GABA-ergic) signaling. Additional information on glutamate vs. GABA concentrations as derived from MRS or behavioral proxies of GABA signaling may help to interpret an individual’s score on the EEG measure. For a stratification biomarker to be predictive of treatment response those differences are critical as it may indicate whether – broadly – a GABA agonist or glutamate (receptor) antagonist may be more likely to be effective for a particular individual. Also a fuller understanding of an individual’s cognitive profile across domains, and the relationship between cognitive strengths and weaknesses is crucial, as individuals with ASD and their families may be more likely to accept future medical treatments if they could be reassured that those strengths, which form an important part of a person’s identity, are not blunted by them.

Longitudinal designs with at least three time points are needed that concurrently track changes in the clinical, neurocognitive, functional, and anatomical trajectories to ascertain the prognostic value of stratification biomarkers. This way, we can begin to model whether a person whose social-communicative skills improve between, say childhood and adolescence has a different neurobiological profile to someone whose symptoms stay the same or worsen. Identification of convergent/divergent pathways into the disorder (risk biomarkers) would be particularly useful to estimate whether a child will develop an ASD before it is clinically manifest and to offer intervention or treatments during early development. Research on risk biomarkers typically employs the high-risk infant sibling design, which capitalizes on the finding that siblings of a child with ASD have a 20-fold increased risk to also developing ASD. So far, the majority of infant-at-risk studies have treated “high-risk” infants as a relatively homogeneous group and only typically stratified by whether or not they developed ASD at age 2–3 years. Identification of “prodromal” subgroups is difficult given that this design is so costly and the number of children who develop ASD in these cohorts is relatively small.

*Validation* of stratification biomarker requires determining their accuracy (i.e., sensitivity, specificity, positive and negative predictive values), plausibility (causal or mechanistically understandable), reliability in relating to a certain clinical endpoint, and reproducibility across clinically relevant settings (53). To increase reproducibility, large-scale consortium studies, such as the IMI-funded EU-AIMS (54) and the NIMH funded Autism Biomarker Consortium-Clinical Trials, are committed to sharing protocols and data.

## Conclusion

The current assumption that ASD involves multiple etiologies and pathophysiological mechanisms makes precision medicine the most promising approach to effective treatments for individuals with the overall umbrella condition. Because in the majority of individuals with ASD (including most with IQ in the normal range) the etiology is currently not known, the approach hinges on a better understanding of whether cellular or molecular mechanisms are shared. The recently failed clinical trials with monogenic forms of ASD point to further obstacles: conceptual and methodological issues linked to translatability from animal models to humans, clinical trial design, placebo effects, and selection of mechanistically plausible, objective outcome measures. Addressing these different factors in the next generation of research studies requires a translatable developmental perspective and multidisciplinary, collaborative initiatives.

## Author Contributions

EL has produced the first and final draft of the paper. DM and WS have commented on and revised several versions of this manuscript.

## Conflict of Interest Statement

WS is an employee of Hoffman-La Roche. EL and DM declare no conflict of interest. The reviewer ED and handling Editor declared their shared affiliation, and the handling Editor states that the process nevertheless met the standards of a fair and objective review.
